# Phenotypic consequences of TLR7-driven interferon and proinflammatory cytokine production in lupus

**DOI:** 10.1186/ar4643

**Published:** 2014-09-18

**Authors:** Westley H Reeves, Haoyang Zhuang, Yuan Xu, Shuhong Han, Hai Wang, Lijun Yang

**Affiliations:** 1 University of Florida, Gainesville, FL, USA

## Background

Although type I interferons (IFN-I) are involved in the pathogenesis of SLE, clinical heterogeneity of the disease is not fully explained by IFN-I overproduction. We investigated the relative importance of IFN-I versus proinflammatory cytokine production downstream of TLR7 in a murine lupus model and in SLE patients.

## Methods

TLR7-mediated experimental lupus was induced in wild-type and knockout mice by pristane and the expression of IFN-I stimulated genes and numbers of plasmacytoid dendritic cells (pDCs) and inflammatory (Ly6C^hi^) monocytes were assessed by PCR and flow cytometry, respectively. Production of TNFα and other proinflammatory cytokines was evaluated by intracellular staining. In parallel, we studied production of these cytokines in the bone marrow (BM) of SLE patients.

## Results

SLE patients' BM exhibited striking death of niche and hematopoietic cells associated with local TNFα overproduction. BM from mice with pristane-induced lupus showed similar abnormalities. TNFα was produced mainly by BM neutrophils, many of which contained phagocytosed nuclear material (LE cells). TNFα production was abolished in TLR7^-/- ^and μmt mice but was unaffected by C3 deficiency. Production was restored in μmt mice by infusing normal plasma, consistent with opsonization of endogenous TLR7 ligands by immunoglobulin. Although autoantibody production and glomerulonephritis are abolished in interferon receptor (IFNAR)^-/- ^mice, both wild-type and IFNAR^-/- ^mice developed anemia and BM hypocellularity following pristane treatment. These manifestations were absent in TLR7^-/- ^and TNFα^-/- ^mice, indicating that the anemia is TNFα mediated. Unexpectedly, although TNF inhibitors can induce lupus manifestations, TNFα^-/- ^mice did not develop autoantibodies spontaneously. However, although IFN-I levels were comparable in untreated TNFα^-/- ^and B6 mice, TNFα^-/- ^mice had increased circulating pDCs and "pDC-like" cells, enhancing their potential to make IFN-I. When treated with pristane, TNFα^-/- ^mice developed more severe lupus than controls with increased levels of anti-Sm/RNP autoantibodies, IFN-I, pDCs, and peritoneal inflammatory (Ly6C^hi^) monocytes.

## Conclusions

Although autoantibodies and glomerulonephritis are TLR7/IFN-I dependent, lupus-associated BM abnormalities were TLR7/TNFα driven, but IFN-I independent. TNFα had a downregulatory effect on pDC numbers and TNFα-deficient mice exhibited an enhanced potential to produce IFN-I in response to TLR7 ligands. Our data suggest that the clinical manifestations of lupus reflect the complex interplay of cells producing IFN-I (causing autoantibodies and nephritis) and TNFα (causing hematologic involvement and arthritis). Engagement of TLR7 by RNA ligands released by dead cells may be a central defect in lupus, whereas the balance of IFN-I versus TNFα production may help determine the disease phenotype.

